# The inhibitory effect of moral emotions on malevolent creativity: Exploring the mediation role of emotional valence and prosocial behavior

**DOI:** 10.3389/fpsyg.2022.945848

**Published:** 2022-08-17

**Authors:** Hongyu Fu, Zhonglu Zhang

**Affiliations:** Department of Psychology in School of Education, Guangzhou University, Guangzhou, China

**Keywords:** malevolent creativity, guilt, gratitude, valence, prosocial behavior

## Abstract

The current study aims to investigate the influence of positive and negative moral emotions (gratitude and guilt) on malevolent creativity by exploring the potential mediation role of valence and prosocial behavior. Using autobiographical recall, three groups of participants developed gratitude, guilt, or neutral emotion, respectively, and then their prosocial behavior and malevolent creativity performance were compared. Results showed that compared with the neutral condition, individuals in the gratitude state experienced more positive emotions with less malevolent creative ideas, but the positive valence pathway had a positive effect on malevolent creativity, indicating the promoting effect of positive emotion on creativity. By contrast, individuals in the guilt state experienced more negative emotions, which result in less malevolent creativity. Gratitude and guilt promoted prosocial behaviors, which did not mediate the effect of gratitude or guilt on malevolent creativity. In short, the results indicate that the positive and negative moral emotions (gratitude and guilt) inhibit malevolent creativity, which is mediated by valence, instead of prosocial behavior.

## Introduction

### Malevolent creativity: Concept and impact factors

Creativity seems a double-edged sword: on the one hand, creativity can be defined as the ability to produce novel products or ideas, which is valuable in solving problems and promoting social progress and innovation (Runco and Jaeger, 2012); on the other hand, when an individual's motive is to harm others and destroy society, the ability of such an idea or product is referred to as malevolent creativity, which is defined as creativity that intentionally harms society, others, or property (Cropley et al., 2008, 2014; Kapoor and Kaufman, 2022). The products of malevolent creativity are so wide-ranging, from everyday fraud to premeditated criminality or terrorist attacks. It is necessary to find ways to curb it. Malevolent creativity is affected by many factors, such as unfair situations, attention bias, impulse control, emotional intelligence, and approach–avoidance motivation (Gill et al., 2013; Harris et al., 2013; Jonason et al., 2014; Gutworth et al., 2016; Gong et al., 2017; Hao et al., 2020), as well as emotion and morality.

Previous studies have indicated that malevolent creativity is closely related to emotion and morality. For instance, researchers have found that anger can promote malevolent creativity in negative emotions (Cheng et al., 2021a,b), and shame can restrain lies (Fan et al., 2019). Although lying belongs to the component of malevolent creativity (Hao et al., 2016), it cannot represent malevolent creativity as it only reflects immoral behavior. Regarding morality, meta-analysis indicates that creativity is positively related to immorality and dark personality (Lebuda et al., 2021; Storme et al., 2021). The moral foundation and the positive correlation between dark personality and creativity are mainly reflected in malevolent creativity (Kapoor and Kaufman, 2021). The malevolent behavior of highly creative people is also regulated by moral reasoning (Zhao et al., 2022). Considering the close relationship between malevolent creativity and emotion, as well as morality, it is meaningful to test whether and how moral emotions influence malevolent creativity.

### Moral emotions and malevolent creativity

Moral emotion refers to the emotion caused by individuals' evaluation of their own or others' behaviors and thoughts according to social norms or codes of conduct (Eisenberg, 2000; Haidt, 2003; Tangney et al., 2007). Moral emotions can be divided into different categories according to different criteria or dimensions. For example, moral emotions can be divided into positive or negative emotions according to valence. According to the criterion of motivational tendency, moral emotions can also be divided into prosocial or antisocial emotions (Haidt, 2003; Rudolph et al., 2013). Interestingly, guilt and gratitude are two types of moral emotions which share common and different features in these two dimensions. From the perspective of motivational tendency, both guilt and gratitude are typical prosocial emotions related to prosocial behavior closely (de Hooge et al., 2007; Ma et al., 2017; Tang et al., 2019). From the perspective of valence, on the one hand, guilt is an unpleasant negative emotional experience that occurs when individuals realize that they have done something that violates moral principles or hurts others and should be responsible for it (Haidt, 2003; de Hooge et al., 2007). On the other hand, gratitude is defined as a complex emotional experience of pleasure generated by individuals when they receive favors (McCullough et al., 2001). Gratitude originates with thankfulness from the behavior of others, which has the function of promoting individuals to care for others and delivering supportive social connections (Grant and Gino, 2010; Ma et al., 2017). Although both are prosocial emotions, gratitude has positive valence and can cause personal pleasure. Guilt has a negative valence and makes one feel bad (de Hooge et al., 2007; Rudolph et al., 2013). Guilt is different from anger and shame, although all three are negative emotions. Anger is a basic emotion, which can promote malevolent creativity by stimulating individual aggression (Cheng et al., 2021a). Guilty and shame are both moral emotions. Shame is triggered by avoiding punishment, so individuals have the motivation to protect their self-image (de Hooge et al., 2007). Guilt is a self-condemning emotion with moral compensation, this refers to the fact that guilt is an emotion that tends to undertake compensatory behavior in self-condemnation of morality, such as clean behavior and prosocial behavior (Zhong and Liljenquist, 2006; Conway and Peetz, 2012; Piazza et al., 2013).

Moral emotions may weaken malevolent creativity: On the one hand, motives and products of malevolent creativity often involve hurting others, aggression, and antisocial behaviors, which are opposite to prosocial behaviors and benevolence, while moral emotions often affect prosocial behaviors and motives (Cropley et al., 2008). Previous meta-analyses indicated that guilt and gratitude are positively correlated with prosocial behavior (Ma et al., 2017; Tang et al., 2019). These suggest that prosocial emotion may be an important factor affecting malevolent creativity. On the other hand, malevolent creativity is related to emotional valence. As the core factor in the construction of emotion, emotional valence is the state in which individuals experience positive or negative emotion (Rasmussen and Berntsen, 2009). For example, anger is a negative emotion, which causes individuals to experience unpleasant feelings (Cheng et al., 2021a). In moral emotions, gratitude can make individuals experience wellbeing and happiness with positive valence, while guilt makes individuals feel bad and depressed with negative valence (de Hooge et al., 2007; Alkozei et al., 2018). The meta-analyses of previous studies on emotion and creativity indicated that positive emotions promote creativity, while negative emotions hinder creativity (Baas et al., 2008; Davis, 2009). In sum, gratitude and guilt may be emotional factors effectively inhibiting malevolent creativity. The pathway may be the effect of gratitude and guilt on prosocial behavior or the effect of emotional valence.

### Current study

To this end, it is largely unknown how moral emotions (e.g., guilt and gratitude) influence malevolent creativity. From the perspective of prosocial emotion and valence, the current study aims to explore the influence of gratitude and guilt on malevolent creativity and whether there is a mediating effect of prosocial behavior or emotional valence. In this study, guilt and gratitude were induced by an autobiographical recall to investigate whether individuals were more likely to engage in prosocial behaviors and less likely to exhibit malevolent creativity under two types of moral emotions and whether prosocial behaviors or emotional valence could inhibit malevolent creativity.

Based on the aforementioned literature, we aim to test the following hypotheses in this study: (1) Gratitude and guilt inhibit the performance of malevolent creativity. (2) Gratitude and guilt make individuals more inclined to engage in prosocial behaviors. (3) Gratitude makes individuals experience positive emotions, while guilt makes individuals experience negative emotions. (4) The inhibition effect of gratitude and guilt on malevolent creativity is mediated by prosocial behavior. (5) Gratitude and guilt inhibit malevolent creativity through different valences.

## Methods

### Participants

A total of 111 participants were recruited for the experiment, including 24 male and 87 female participants (*M*_*age*_ = 19.50 ± 1.07 years). The participants were randomly assigned to the guilt, neutral, or gratitude group, with a balanced gender (29 women and eight men in each group). In total, two participants had their data removed (one reported being honest and guilt-free, and the other participant wrote too little in the autobiographical recall stage, which is far from meeting our minimum word count requirement). We chose the *F* test in G^*^Power 3.1.9.7. The statistical test power of this study was analyzed by *post hoc* estimation. We set the effect size to large (*f* = 0.40) and the significance level at α = 0.05. The result shows that power (*1-*β) = 0.97 > 0.80, which indicates that the sample size of this study meets the statistical requirements.

### Materials

#### Emotion induction and treatment of the control group

The autobiographical recall paradigm was introduced to induce different types of moral emotions (Parkinson and Illingworth, 2009; Stearns and Parrott, 2012; Peng et al., 2018; Cheng et al., 2021a). The gratitude group was asked to recall an event they were grateful for, the guilt group was asked to recall a bad thing they felt guilty about, and the neutral group was asked to complete a control task (keeping a detailed diary of their day). The participants were required to write no <150 words within 15 min. They filled out their memories on a given paper sheet.

#### Emotional manipulation check and valence measurement

The Chinese version of the PANAS scale was used for the pre-test and post-test (Qiu et al., 2008). The scale included nine positive and nine negative emotional experience descriptions. The participants were asked to rate how much they currently experienced the emotions described by these words on a five-point scale ranging from 1 (*very slightly)* to 5 (*extremely*). In the study, the positive valence was the cumulative score of the positive affect subscale in PANAS (Cronbach's α = 0.92), and the negative valence was the cumulative score of the negative affect subscale in PANAS (Cronbach's α = 0.90).

#### Measurement of prosocial behavior

Using the scenario material compiled by Xia et al. (2021), two questions represent the willingness and energy of individuals to participate in prosocial behaviors, which can effectively measure prosocial behaviors (Grant and Gino, 2010). The contents are as follows:

Now, the school will organize voluntary activities in welfare homes and nursing homes for the elderly. At the request of the hospital, volunteers should concentrate on accompanying the elderly or children in the process of volunteering. Therefore, the school volunteer association requires volunteers to participate in the activity within 3 h of the activity, try not to use mobile phones and other communication equipment to do irrelevant things, and concentrate on the content of the activity. (1) How likely do you will volunteer for the voluntary activity? (1-definitely will not, 7-definitely will). (2) If you are willing to volunteer, how many days will you spend volunteering 7 days a week? (Participants were asked to choose a number in the 0,7 days of the week).

The total score after the standardization (Z-score) of the two questions is used as the indicator of prosocial behaviors (total score = Q1 (Z-score) + Q2 (Z-score)).

#### Measurement of malevolent creativity

The malevolent creativity task (MCT) was used to measure the performance of individuals' malevolent creativity (Hao et al., 2020). The MCT written by Hao et al. (2020) is as follows: “Ming (a name) was walking on his way 1 day. Wei (a name) was running in a hurry and bumped into Ming, and Ming's computer dropped to the ground and broke. Wei criticized Ming and ran off without saying that he was sorry, which made Ming very angry.” Participants were asked to generate as many creative ideas as possible to help Ming take revenge on Wei without being discovered within 5 min. The performance of malevolent creativity was measured by fluency, originality, and harmfulness. The total score of malevolent creativity was calculated by adding the standardized score (Z-score) of fluency, originality, and harmfulness [Total score = Fluency (Z-score) + Originality (Z-score) + Harmfulness(Z-score)]. Fluency refers to the number of valid ideas generated. A total of four raters evaluated each idea separately based on whether it is a malevolent idea and calculated the fluency (ICC = 0.91). Originality refers to the novelty and uniqueness of generating ideas. A total of four raters assigned each idea a score for originality based on how often it appeared in the sample. Specifically, 2, 1, and 0 points are assigned to the ideas whose frequency is ≤ 1%, 1% ~ 5%, or > 5%, respectively (Hao et al., 2020; Cheng et al., 2021a). The final originality score (ICC = 0.81) was taken as the total originality score of all the ideas of each participant. Harmfulness refers to the hurtful degree of the generated ideas. Each of the four raters had a 5-point assessment on the harmfulness of each idea (ICC = 0.89). The score of fluency, originality, and harmfulness of each idea was averaged across the four raters.

### Procedure

The participants completed the emotional pre-test (PANAS). Then, the participants were asked to complete the autobiographical recall task in the group. After emotion induction, the participants completed emotional the post-test (PANAS) and prosocial situation tasks. The participants were then asked to complete a five-minute malevolent creativity task in a written form.

## Results

### Validation of emotion induction

In order to validate emotion induction, paired sample *t*-tests were used, respectively, on the pre-test and post-test of emotions for each of the three groups (see Table 1). The results are as follows: (1) For the guilt group, the guilt scores of the post-test (*M* = 3.31, *SD* = 0.80) were significantly higher than those in the pre-test (*M* = 1.40, *SD* = 0.76), *t*_(34)_ = −11.20, *p* < 0.001. (2) For the gratitude group, the gratitude score of the post-test (*M* = 4.05, *SD* = 0.88) was significantly higher than that in pre-test (*M* = 2.41, *SD* = 1.14), *t*_(36)_ = −8.01, *p* < 0.001. (3) There was no significant difference in the guilt or gratitude score between the pre-test of guilt (*M*_(*guilt*)_ = 1.27, *SD* = 0.56; *M*_(*gratitude*)_ = 2.38, *SD* = 1.06) and the post-test (*M*_(*guilt*)_ = 1.35, *SD* = 0.72; *M*_(*gratitude*)_ = 2.22, *SD* = 1.06) in the neutral group (*t*_(*guilt*)_ (36) = −0.77, *p* = 0.446; *t*_(*gratitude*)_ (36) = −0.85, *p* = 0.404). This indicated that emotion induction was successfully manipulated.

**Table 1:** Descriptive statistics of emotions (*M* ± *SD*) and paired sample *t*-test.

**Group**	**Pre-test**	**Post-test**	***T***	***p***
Guilt group				
Guilt	1.40 ± 0.76	3.31 ± 0.80	−11.203	<0.001
Gratitude group				
Gratitude	2.41 ± 1.14	4.05 ± 0.88	−8.010	<0.001
Neutral group				
Gratitude	2.38 ± 1.06	2.22 ± 1.06	0.845	0.404
Guilt	1.27 ± 0.56	1.35 ± 0.72	−0.770	0.446

### The effect of gratitude and guilt on emotional valence

A one-way MANOVA using emotion (guilt, gratitude, and control) as the between-group factor was conducted on the pre-test data of positive and negative emotions, with *Box's M* = 17.63, *F* = 2.86, *p* = 0.009. The results showed that the covariance matrices of these dependent variables were not homogeneous and the data did not fit MANOVA. Thus, one-way ANOVAs using emotion as the between-group factor were conducted on positive and negative emotions, respectively. The results showed that in the pre-test, there was neither any significant difference in positive emotions [*F*_(2, 106)_ = 0.12, *p* = 0.892] nor any significant difference in the negative emotions [*F*_(2, 106)_ = 1.24, *p* = 0.294] among the three groups.

A one-way MANOVA using emotion as the between-group factor was conducted on the post-test of positive and negative emotions, showing *Box's M* = 10.40, *F* = 1.69, *p* = 0.120. The results showed that the covariance matrices of these dependent variables were homogeneous, and the data are suitable for MANOVA. The main effect of different emotional groups on positive emotions was significant, *F*_(2, 106)_ = 17.06, *p* < 0.001, eta^2^ = 0.24. *Post hoc* tests showed that the gratitude group (*M* = 29.32, *SD* = 8.45) was higher than that neutral group (*M* = 23.97, *SD* = 7.68, *p* = 0.004, Cohen's *d* = 8.07) and guilt group (*M* = 18.54, *SD* = 7.29, *p* < 0.001, Cohen's *d* = 7.91), and the guilt group was lower than the neutral group (*p* = 0.004, Cohen's *d* = 7.49). The main effect of different emotional groups on negative emotions was significant, *F*_(2, 106)_ = 37.43, *p* < 0.001, eta^2^ = 0.41. *Post hoc* tests showed that the guilt group (*M* = 18.37, *SD* = 4.58) was higher than the gratitude group (*M* = 11.57, *SD* = 3.44, *p* < 0.001, Cohen's *d* = 4.03) and neutral emotion group (*M* = 11.32, *SD* = 3.64). *p* < 0.001, Cohen's *d* = 4.12), and there was no significant difference between the neutral group and the gratitude group (*p* = 0.789).

### The influence of gratitude and guilt on prosocial behavior

A one-way ANOVA using emotion as the between-group factor was conducted on prosocial behavior. The results showed that the main effect of emotion on prosocial behavior was significant, *F*_(2, 106)_ = 3.90, *p* = 0.023, eta^2^ = 0.07. *Post hoc* tests showed that the neutral group (*M* = −0.60, *SD* = 1.98) was lower than the gratitude group (*M* = 0.309, *SD* = 1.33, *p* = 0.017, Cohen's *d* = 1.69) and guilt group (*M* = 0.306, *SD* = 1.41). *p* = 0.018, Cohen's *d* = 1.73), and there was no significant difference between the guilt group and the gratitude group (*p* = 0.993).

### The influence of gratitude and guilt on malevolent creativity

**Figure 1:**
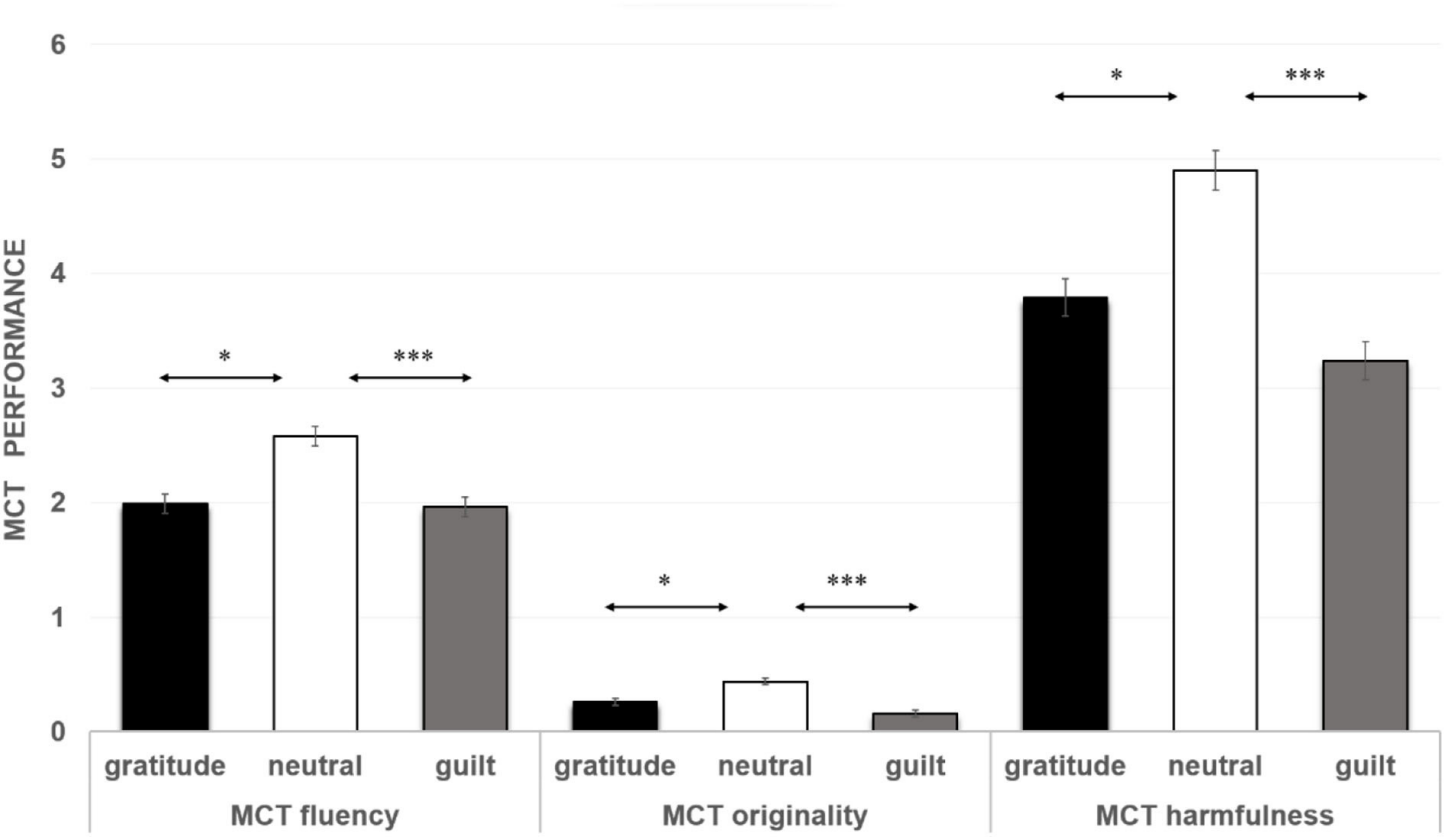
Malevolent creativity task performance in different emotion groups in experiment. The error bars represent standard errors. *p < *0.05*, ***p < *0.001*.

A one-way MANOVA using emotion as the between-group factor was conducted on the MCT fluency, originality, and harmfulness of MCT, *Box's M* = 9.51, *F*=0.76, *p* = 0.693. The results showed that the covariance matrices of each dependent variable are homogeneous, and the data are suitable for multivariate variance analysis. The results showed that the main effect of moral emotion on MCT fluency was significant, *F*_(2, 106)_ = 4.43, *p* = 0.014, eta^2^ = 0.08. *Post hoc* tests showed that the neutral group (*M* = 2.58, SD = 1.06) was higher than the gratitude group (*M* = 1.99, *SD* = 0.96, *p* = 0.014, Cohen's *d* = 1.00) and guilt group (*M* = 1.96, *SD* = 1.00, *p* = 0.010, Cohen's *d* = 1.03), and there was no significant difference between the guilt group and the gratitude group (*p* = 0.879). The main effect of moral emotion on MCT originality was significant, *F*_(2, 106)_ = 5.87, *p* = 0.004, eta^2^ = 0.10. *Post hoc* tests showed that the neutral emotion group (*M* = 0.44, *SD* = 0.40) was higher than the gratitude group (*M* = 0.26, *SD* = 0.35, *p* = 0.029, Cohen's *d* = 0.37) and guilt group (*M* = 0.16, *SD* = 0.31, *p* = 0.001, Cohen's *d* = 0.36), and there was no significant difference between the guilt group and the gratitude group (*p* = 0.237). The main effect of moral emotion on MCT harmfulness was significant, *F*_(2, 106)_ = 6.37, *p* = 0.002, eta^2^ = 0.11. *Post hoc* tests showed that the neutral emotion group (*M* = 4.90, *SD* = 2.06) was higher than the gratitude group (*M* = 3.79, *SD* = 2.02, *p* = 0.020, Cohen's *d* = 2.04) and guilt group (*M* = 3.24, *SD* = 1.95, *p* < 0.001, Cohen's *d* = 2.00), and there was no significant difference between the guilt group and the gratitude group (*p* = 0.251) (see Figure 1).

**Figure 2:**
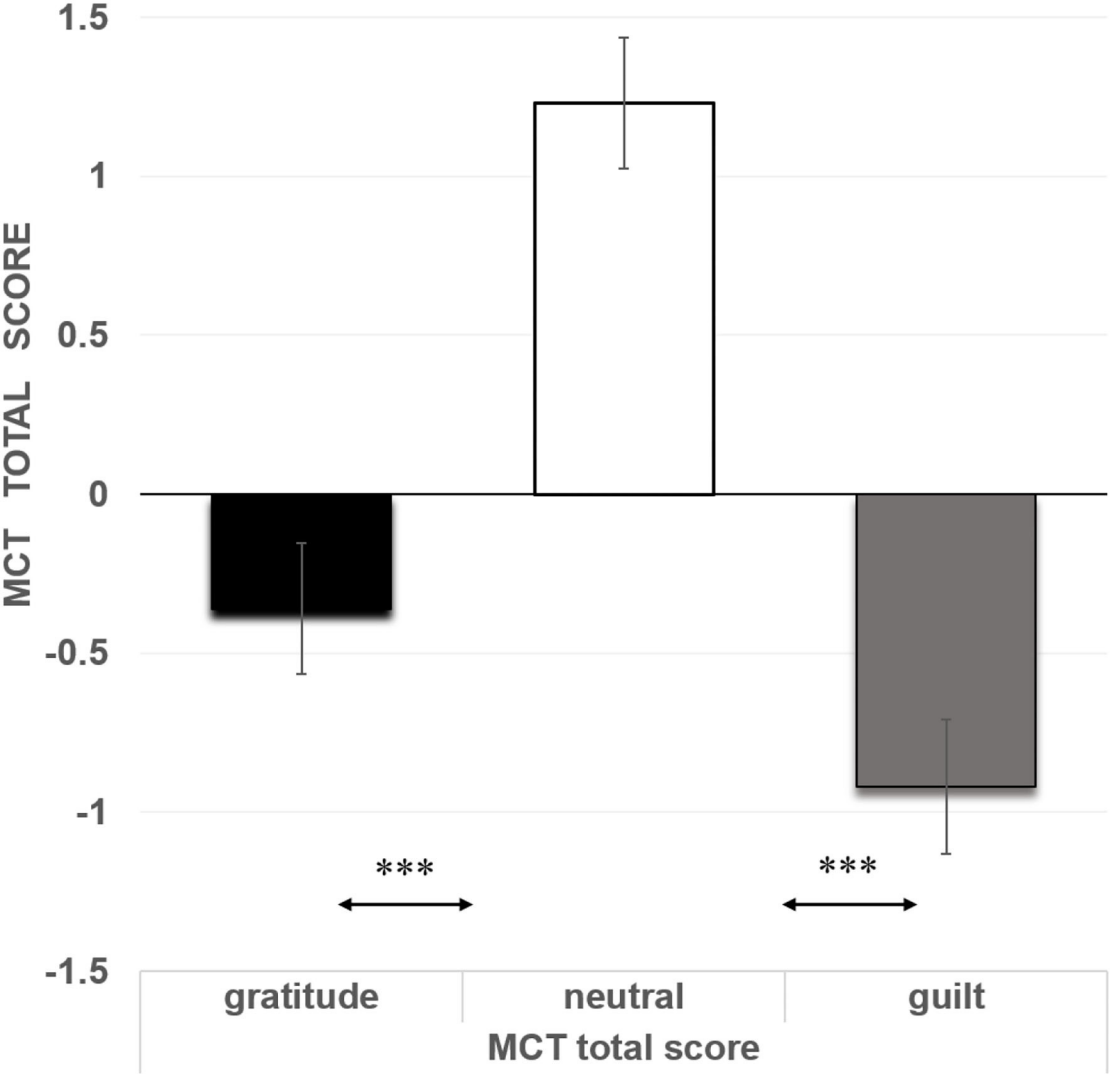
Malevolent creativity The task total score in different emotion groups in experiment. The error bars represent standard errors. ***p < *0.001*.

A one-way ANOVA using moral emotion as the between-group factor was conducted on the MCT total score (standardized score). The main effect of moral emotion on the MCT total score was significant, *F*_(2, 106)_ = 7.38, *p* = 0.001, eta^2^ = 0.12. *Post hoc* tests showed that the neutral emotion group (*M* = 1.23, *SD* = 2.67) was higher than the gratitude group (*M* = −0.36, *SD* = 2.44, *p* = 0.007, Cohen's *d* = 2.55) and guilt group (*M* = −0.92, *SD* = 2.28, *p* < 0.001, Cohen's *d* = 2.49), and there was no significant difference between the guilt group and the gratitude group (*p* = 0.336) (see Figure 2).

### The mediation analyses of emotional valence and prosocial behavior

**Figure 3:**
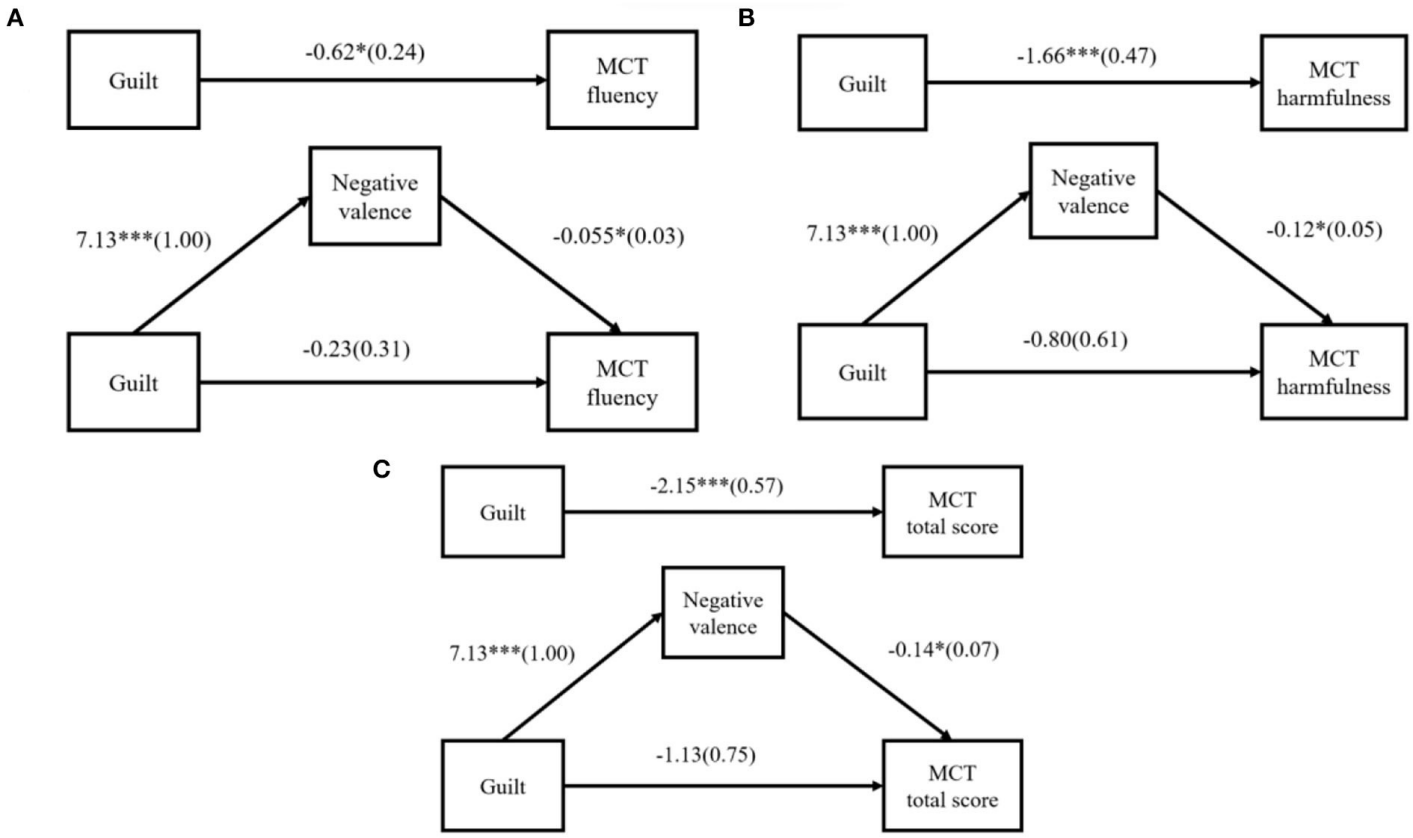
**(A–C)** Mediation analysis using negative valence as the mediator. *p < *0.05*, ***p < *0.001*. The coefficient is non-standard coefficient, and the standard error is in ().

To further test whether the effect of guilt on malevolent creativity was mediated through emotional valence, and the PROCESS was used to perform boostrap-based mediation effect analysis (Hayes, 2013; Hayes and Preacher, 2014), with post-test negative emotions as the mediator, the independent variables coded as dummy variables (1 = guilty, 0 = neutral), and fluency, originality, harmfulness, and total score in MCT as dependent variables. The sample size was 5,000, and the confidence interval was 95%. The results showed the direct effect of guilt on MCT fluency was not significant, *b* = −0.23, *p* = 0.462, CI = [−0.86, 0.39], while the indirect effect of emotion valence on MCT fluency was significant, *b* = −0.39, CI = [−0.80, −0.02] (see Figure 3A). There was neither a significant direct effect of guilt on MCT originality (*b* = −0.20, *p* = 0.080, CI = [−0.42, 0.02]) nor an indirect effect of emotional valence on MCT originality (*b* = −0.09, CI = [−0.22, 0.02]). The direct effect of guilt on MCT harmfulness was not significant, *b* = −0.80, *p* = 0.193, CI = [−2.01, 0.41], while the indirect effect of emotional valence on MCT harmfulness was significant, *b* = −0.86, CI = [−1.74, −0.14] (see Figure 3B). The direct effect of guilt on the MCT total score was not significant, *b* = −1.13, *p* = 0.138, CI = [−2.64, 0.37], while the indirect effect of emotional valence on the MCT total score was significant, *b* = −1.02, CI = [−2.02, −0.13] (see Figure 3C). In short, negative valence completely mediated the inhibition effect of guilt on the overall performance of malevolent creativity.

The mediation analysis using prosocial behavior as the mediator showed that guilt had a significant direct effect on MCT fluency, *b* = −0.63, *p* = 0.015, CI = [−1.14, −0.12], while prosocial behavior had no significant indirect effect on MCT fluency, *b* = 0.01, CI = [−0.13, 0.16]. The direct effect of guilt on MCT originality was significant, *b* = −0.30, *p* = 0.001, CI = [−0.47, −0.12], while the indirect effect of prosocial behavior on MCT originality was not significant, *b* = 0.02, CI = [−0.026, 0.076]. The direct effect of guilt on MCT harmfulness was significant, *b* = −1.68, *p* = 0.001, CI = [−2.66, −0.69], while the indirect effect of prosocial behavior on MCT harmfulness was not significant, *b* = 0.02, CI = [−0.23, 0.36]. The direct effect of guilt on the MCT total score was significant, *b* = −2.21, *p* < 0.001, CI = [−3.42, −0.99], while the indirect effect of prosocial behavior on the MCT total score was not significant, *b* = 0.06, CI = [−0.22, 0.44]. These results suggest that prosocial behavior has no mediating effect on the effect of guilt on malevolent creativity (see Table 2).

**Table 2:** Mediation analyses of prosocial behavior in guilt (block 1) and in gratitude (block 2).

**Block 1**	**Guilt**	***B* of**	**95% *CI* of**	***B* of**	**95% *CI* of**
		**direct effect**	**direct effect**	**indirect effect**	**indirect effect**
	MCT fluency	−0.63*	[−1.14, −0.12]	0.006	[−0.13, 0.16]
	MCT originality	−0.30**	[−0.47, −0.12]	0.02	[−0.03, 0.09]
	MCT harmfulness	−1.68**	[−2.66, −0.69]	0.02	[−0.03, 0.08]
	MCT total score	−2.21***	[−3.42, −0.99]	0.06	[−0.22, 0.44]
**Block 2**	**Gratitude**	***B*** **of**	**95%** ***CI*** **of**	***B*** **of**	**95%** ***CI*** **of**
		**direct effect**	**direct effect**	**indirect effect**	**indirect effect**
	MCT fluency	−0.60*	[−1.08, −0.11]	0.01	[−0.10, 0.14]
	MCT originality	−0.20*	[−0.38, −0.02]	0.02	[−0.02, 0.08]
	MCT harmfulness	−1.17*	[−2.16, −0.18]	0.06	[−0.18, 0.37]
	MCT total score	−1.68**	[−2.91, −0.44]	0.09	[−0.17, 0.47]

* p < 0.05,

** p < 0.01,

*** p < 0.001.

**Figure 4:**
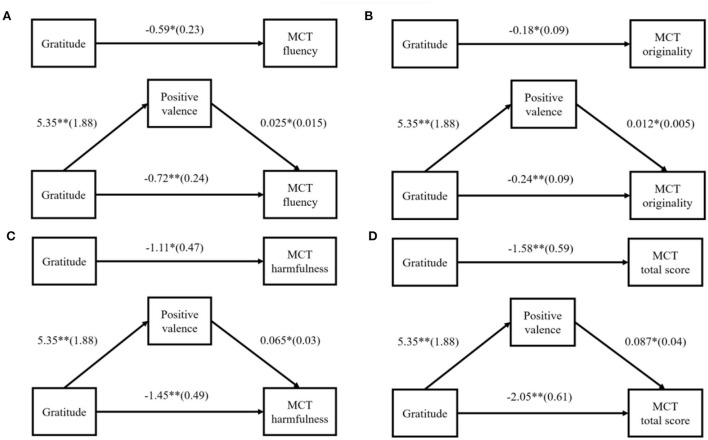
**(A–D)** Mediation analysis using positive valence as the mediator. *p < *0.05*, **p < *0.01*. The coefficient is non-standard coefficient, and the standard error is in ().

A similar mediating effect analysis was conducted for the gratitude group. The independent variables coded as dummy variables (1 = grateful, 0 = neutral), with fluency, originality, harmfulness, and total score in MCT as dependent variables. The mediation analysis using post-test positive emotions as mediator showed that the direct effect of gratitude on MCT fluency was significant, *b* = −0.72, *p* = 0.004, CI = [−1.21, −0.23], while the indirect effect of emotion valence on MCT fluency was significant, *b* = 0.13, CI = [0.007, 0.35] (see Figure 4A). The direct effect of gratitude on MCT originality was significant, *b* = −0.25, *p* = 0.007, CI = [−0.43, −0.07], while the indirect effect of emotional valence on MCT originality was significant, *b* = 0.06, CI = [0.003, 0.15] (see Figure 4B). The direct effect of gratitude on MCT harmfulness was significant, *b* = −1.45, *p* = 0.004, CI = [−2.43, −0.48], while the indirect effect of emotional valence on MCT harmfulness was significant, *b* = 0.35, CI = [0.04, 0.85] (see Figure 4C). The direct effect of gratitude on the MCT total score was significant, *b* = −2.05, *p* = 0.001, CI = [−3.26, −0.84], and the indirect effect of emotional valence on the MCT total score was significant, *b* = 0.47, CI = [0.06, 1.08] (see Figure 4D). In short, positive valence partly mediated the effect in the inhibition of the overall performance of malevolent creativity by gratitude.

The mediation analysis using prosocial behavior as mediator showed that the direct effect of gratitude on MCT fluency was significant (*b* = −0.60, *p* = 0.018, CI = [−1.08, −0.11]), while the indirect effect of prosocial behavior on MCT fluency was not significant (*b* = 0.01, CI = [−0.10, 0.14]. The direct effect of gratitude on MCT originality was significant (*b* = −0.20, *p* = 0.028, CI = [−0.38, −0.02]), while the indirect effect of prosocial behavior on MCT originality was not significant (*b* = 0.02, CI = [−0.02, 0.08]). The direct effect of gratitude on MCT harmfulness was significant (*b* = −1.17, *p* = 0.021, CI = [−2.16, −0.18]), while the indirect effect of prosocial behavior on MCT harmfulness was not significant (*b* = 0.06, CI = [−0.18, 0.37]). The direct effect of gratitude on the MCT total score was significant (*b* = −1.68, *p* = 0.008, CI = [−2.91, −0.44]), while the indirect effect of prosocial behavior on the MCT total score was not significant (*b* = 0.09, CI = [−0.17, 0.47]). These results suggest that prosocial behavior has no mediating effect on the effect of gratitude on malevolent creativity (see Table 2).

## Discussion

Overall, this study tests the potential mediation roles of valence and prosocial behavior in the influence of moral emotion (gratitude and guilt) on malevolent creativity. The results mainly revealed that gratitude and guilt inhibit the performance of malevolent creativity through the mediation role of emotion valence, instead of prosocial behavior.

### Gratitude and guilt: Two prosocial moral emotions with different valences

Previous meta-analyses have proved that guilt and gratitude can promote prosocial behavior (Ma et al., 2017; Tang et al., 2019). Guilt is a negative emotion, while gratitude is a positive emotion (Tangney et al., 2007; Rudolph et al., 2013). Consistently, the current study provides evidence that gratitude and guilt are positive and negative prosocial moral emotions, respectively. Specifically, the results showed that first, relative to neutral conditions, individuals in gratitude conditions experience higher positive emotions, and individuals in guilt conditions experience higher negative emotions; second, both gratitude and guilt make individuals more inclined to engage in prosocial behaviors.

### The inhibitory effect of gratitude and guilt on malevolent creativity: The role of prosocial behavior

More importantly, we revealed that although with different valences, both gratitude (positive moral emotion) and guilt (negative moral emotion) inhibit malevolent creativity by decreasing performance. However, we did not find any significant mediating role of prosocial behavior. One reason may be that we measure prosocial behavior in the study, instead of motivation, which has been demonstrated to be closely related to malevolent creativity (Cropley et al., 2008; Hao et al., 2020). Prosocial behavior is a behavioral result, as a product variable similar to malevolent creativity. Another reason may be due to moral compensation. This study verified that individuals are more inclined to engage in prosocial behaviors under the experience of guilt (Ma et al., 2017), and malevolent creativity can be inhibited. As moral compensation refers to that one tends to do compensation behavior in self-condemnation of morality, such as clean behavior and prosocial behavior (Zhong and Liljenquist, 2006; Piazza et al., 2013), we believe that less malevolent behavior is another manifestation of moral compensation. Guilt increases prosocial behaviors and decreases malevolent creativity directly due to moral compensation, thereby prosocial behaviors and malevolent creativity are parallel, instead of mediated. Gratitude can promote prosocial behaviors and inhabit malevolent creativity, which may be related to attentional bias. Individuals with gratitude generate attention to positive events in social interactions, which enhance prosocial behavior and reduce malevolent motivation (Alkozei et al., 2018).

### The inhibitory effect of gratitude and guilt on malevolent creativity: The role of emotional valence

Moreover, we revealed that emotional valence mediates the inhibition effect of gratitude and guilt on malevolent creativity. Specifically, gratitude inhibited the overall performance of malevolent creativity and was reflected in originality, fluency, and harmfulness. However, gratitude has a positive effect on the performance of malevolent creativity through the pathway of positive valence, thus weakening the total effect of gratitude inhabiting malevolent creativity. Therefore, the influence of positive valence on gratitude inhibiting malevolent creativity is not a simple intermediary effect but a suppression effect. It may be the reason for the promotion effect of positive emotion on creative thinking as malevolent creativity belongs to creative thinking (Baas et al., 2008; Davis, 2009). Guilt inhibits malevolent creativity in two dimensions: fluency and harmfulness, but not in originality. Moreover, the inhibition effect of guilt on malevolent creativity is mainly mediated by negative valence, which not only results from the inhibition effect of the negative valence of emotion on creative thinking (Davis, 2009). Also, it may be related to individual motivation factors and emotion-related cognitive processing.

### The mechanism of the inhibition effect of moral emotions on malevolent creativity: Potential accounts

On this basis, the mechanism of gratitude and guilt affecting malevolent creativity is worth further exploring. From the perspective of the directivity of emotions, guilt is a self-conscious emotion, while gratitude is other-oriented (Rudolph et al., 2013). Therefore, guilt inhibits malevolent creativity due to self-consciousness monitoring and cognitive control of self-behavior (de Hooge et al., 2007). Gratitude may be related to individual attention bias (Grant and Gino, 2010); if individuals tend to focus on the bright side of others or society and positive events related to their own, they lose the tendency of malevolent motives. From the perspective of emotional valence, guilt belongs to negative emotion, whereas gratitude belongs to positive emotion. Malevolent creativity is part of creative thinking. The previous meta-analysis shows that positive emotion promotes creative thinking, while negative emotion inhibits creative thinking (Baas et al., 2008; Davis, 2009), and this study verifies this conclusion. Therefore, the inhibition effect of guilt on malevolent creativity is derived from the inhibition effect of negative valence on creative thinking. The ego depletion and the occupation of cognitive resources caused by the negative valence of guilt may be the reasons for the inhibition of malevolent creativity. The promoting effect of positive emotion on creative thinking is the same as the effect of positive valence in gratitude on malevolent creativity, although gratitude still inhibits malevolent creativity. The reason may be associated with motivation or cognitive control. As shown in the results, gratitude gave individuals more intention to be prosocial, rather than showing malevolent creativity. In that case, individuals with gratitude will produce prosocial motivation, and malevolent behavior under this motivation may cause individual cognitive control and motivation conflict, thus inhibiting malevolent creativity. From the perspective of moral emotion, gratitude and guilt are associated with moral reasoning and judgment (Haidt, 2003; Ma et al., 2017; Tang et al., 2019). Such cognitive processing may inhibit malevolent creativity. Therefore, the inhibition of malevolent creativity by gratitude and guilt also involves higher cognitive processing.

### Implications

This study has certain implications, theoretically and practically. Theoretically, it enriches the research on the influencing factors of malevolent creativity. From the perspective of emotion and creativity, it is essentially a collision between moral emotion and immorality of creativity. Practically, it provides a method to suppress malevolent creativity, through guilt or gratitude. Meanwhile, this study also proves that moral emotions reduce the dark side of creativity in potential threats and social harm effectively.

### Limitations and further study

Several limitations should be noted in this study. First, considering the particularity of moral emotions and the ecological validity of emotional induction, this study only adopted an autobiographical recall task to induce emotions. There are other methods to induce gratitude and guilt emotions, such as audio-visual combination and situational integration. Second, this study only discusses the influence of gratitude and guilt on malevolent creativity but does not explore other moral emotions. There are more than 20 kinds of moral emotions and different classification perspectives (Rudolph et al., 2013). Future studies can explore how more specific moral emotions affect individuals' malevolent creativity from multiple perspectives. In addition, this study is focused on the research category of malevolent creativity, not involving general creativity and benevolent creativity, because it was more focused on the study of gratitude and guilt on the inhibition mechanism of malevolent creativity. Although this study found that emotional valence is guilty and grateful to the path of malevolent creativity, it needs further study to reveal the psychological mechanism. In addition, fNIRS and fMRI can be combined to further explore the cognitive neural mechanism of the impact of moral emotion on malevolent creativity.

## Data availability statement

The raw data supporting the conclusions of this article will be made available by the authors, without undue reservation.

## Ethics statement

The studies involving human participants were reviewed and approved by Guangzhou University Institutional Review Board. The patients/participants provided their written informed consent to participate in this study.

## Author contributions

HF and ZZ led writing, conceptualization, and methodology. HF oversaw data analyses and led data analyses. ZZ provided reviews. Both authors contributed to the article and approved the submitted version.

## Funding

This study was supported by grants from the 18^th^ Challenge Cup Academic Science and Technology Work Competition, a Guangzhou University-established project for school-level work (Grant No. 2022TZBPHC1115) and Guangzhou education scientific research project (Grant No. 202113612).

## Conflict of interest

The authors declare that the research was conducted in the absence of any commercial or financial relationships that could be construed as a potential conflict of interest.

## Publisher's note

All claims expressed in this article are solely those of the authors and do not necessarily represent those of their affiliated organizations, or those of the publisher, the editors and the reviewers. Any product that may be evaluated in this article, or claim that may be made by its manufacturer, is not guaranteed or endorsed by the publisher.

## References

[B1] Alkozei A. Smith R. Killgore W. D. (2018). Gratitude and subjective wellbeing: a proposal of two causal frameworks. J. Happiness Stud. 19, 1519–1542. 10.1007/s10902-017-9870-1

[B2] Baas M. De Dreu C. K. Nijstad B. A. (2008). A meta-analysis of 25 years ofmood-creativity research: Hedonic tone, activation, or regulatory focus? Psychol. Bull. 134, 779–806. 10.1037/a0012815 18954157

[B3] Cheng R. Lu K. Hao N. (2021b). The effects of anger on different forms of malevolent creative performance. J. Psychol. Sci. 44, 1336–1345. 10.16719/j.cnki.1671-6981.20210608

[B4] Cheng R. Lu K. L. Hao N. (2021a). The effect of anger on malevolent creativity and strategies for its emotion regulation. Acta Psychol. Sin. 53, 847–860. 10.3724/SP.J.1041.2021.00847

[B5] Conway P. Peetz J. (2012). When does feeling moral actually make you a better person? Conceptual abstraction moderates whether past moral deeds motivate consistency or compensatory behavior. Pers. Soc. Psychol. Bull. 38, 907–919. 10.1177/0146167212442394 22492550

[B6] Cropley D. H. Kaufman J. C. Cropley A. J. (2008). Malevolent creativity: a functional model of creativity in terrorism and crime. Creat. Res. J. 20, 105–115. 10.1080/10400410802059424

[B7] Cropley D. H. Kaufman J. C. White A. E. Chiera B. A. (2014). Layperson perceptions of malevolent creativity: The good, the bad, and the ambiguous. Psychol. Aesthet. Creat. Arts 8, 400–412. 10.1037/a0037792

[B8] Davis M. A. (2009). Understanding the relationship between mood and creativity: A meta-analysis. Org. Behav. Hum. Decis. Processes 108, 25–38. 10.1016/j.obhdp.2008.04.00122409506

[B9] de Hooge I. E. Zeelenberg M. Breugelmans S. M. (2007). Moral sentiments and cooperation: Differential influences of shame and guilt. Cogn. Emot. 21, 1025–1042. 10.1080/02699930600980874

[B10] Eisenberg N. (2000). Emotion, regulation, and moral development. Annu. Rev. Psychol. 51, 665–697. 10.1146/annurev.psych.51.1.665 10751984

[B11] Fan W. Ren M. Xiao J. Jian Z. Du X. Fu X. (2019). The influence of shame on deceptive behavior: The role of self-control. Acta Psychol. Sin. 51, 992–1006 10.3724/SP.J.1041.2019.00992

[B12] Gill P. Horgan J. Hunter S. T. Cushenbery L. D. (2013). Malevolent creativity in terrorist organizations. J. Creat. Behav. 47, 125–151. 10.1002/jocb.28

[B13] Gong Z. Peng Y. Wang X. Liu C. (2017). The characteristics of attentional bias and impulsive control in highly malevolent creative people. Chin. J. Clin. Psychol. 25, 613–617. 10.16128/j.cnki.1005-3611.2017.04.005

[B14] Grant A. M. Gino F. (2010). A little thanks goes a long way: Explaining why gratitude expressions motivate prosocial behavior. J. Pers. Soc. Psychol. 98, 946. 10.1037/a0017935 20515249

[B15] Gutworth M. B. Cushenbery L. Hunter S. T. (2016). Creativity for deliberate harm: malevolent creativity and social information processing theory. J. Creat. Behav. 52, 305–322. 10.1002/jocb.155

[B16] Haidt J. (2003). “The moral emotions,” in Handbook of Affective Sciences, eds R. J. Davidson, K. R. Scherer, and H. H. Goldsmith (Oxford: Oxford University Press), 852–870.

[B17] Hao N. Qiao X. Cheng R. Lu K. Tang M. Runco M. A. (2020). Approach motivational orientation enhances malevolent creativity. Acta Psychol. 203, 102985. 10.1016/j.actpsy.2019.102985 31863973

[B18] Hao N. Tang M. Jing Y. Wang Q. Runco M. A. (2016). A new tool to measure malevolent creativity: the malevolent creativity behavior scale. Front. Psychol. 7, 682. 10.3389/fpsyg.2016.00682 27242596 PMC4870273

[B19] Harris D. J. Reiter-Palmon R. Kaufman J. C. (2013). The effect of emotional intelligence and task type on malevolent creativity. Psychol. Aesthet. Creat. Arts 7, 237–244. 10.1037/a0032139

[B20] Hayes A. F. (2013). Introduction to Mediation, Moderation, and Conditional Process Analysis: A Regression-Based Approach. New York, NY: The Guilford Press. 10.1111/jedm.12050

[B21] Hayes A. F. Preacher K. J. (2014). Statistical mediation analysis with a multicategorical independent variable. Br. J. Math. Stat. Psychol. 67, 451–470. 10.1111/bmsp.12028 24188158

[B22] Jonason P. K. Lyons M. Baughman H. M. Vernon P. A. (2014). What a tangled web we weave: the dark triad traits and deception. Pers. Individ. Differ. 70, 117–119. 10.1016/j.paid.2014.06.038

[B23] Kapoor H. Kaufman J. C. (2021). Unbound: The relationship among creativity, moral foundations, and dark personality. J. Creat. Behav. 56, 182–193. 10.1002/jocb.523

[B24] Kapoor H. Kaufman J. C. (2022). The evil within: The AMORAL model of dark creativity. Theory Psychol. 32, 467–490. 10.1177/09593543221074326

[B25] Lebuda I. Figura B. Karwowski M. (2021). Creativity and the dark triad: a meta-analysis. J. Res. Pers. 92, 104088. 10.1016/j.jrp.2021.104088

[B26] Ma L. K. Tunney R. J. Ferguson E. (2017). Does gratitude enhance prosociality?: A meta-analytic review. Psychol. Bull. 143, 601–635. 10.1037/bul0000103 28406659

[B27] McCullough M. E. Kilpatrick S. D. Emmons R. A. Larson D. B. (2001). Is gratitude a moral affect? Psychol. Bull. 127, 249–266. 10.1037//0033-2909.127.2.249 11316013

[B28] Parkinson B. Illingworth S. (2009). Guilt in response to blame from others. Cogn. Emot. 23, 1589–1614. 10.1080/02699930802591594

[B29] Peng C. Nelissen R. M. A. Zeelenberg M. (2018). Reconsidering the roles of gratitude and indebtedness in social exchange. Cogni. Emot. 32, 760–772. 10.1080/02699931.2017.1353484 28718342

[B30] Piazza J. Russell P. S. Sousa P. (2013). Moral emotions and the envisaging of mitigating circumstances for wrongdoing. Cogn. Emot. 27, 707–722. 10.1080/02699931.2012.736859 23098124

[B31] Qiu L. Zheng X. Wang Y. F. (2008). Revision of the positive affect and negative affect scale. Chin. J. Appl. Psychol. 14, 249–254. Available online at: https://bit.ly/3bhnijD

[B32] Rasmussen A. S. Berntsen D. (2009). Emotional valence and the functions. Mem. Cogn. 37, 477–492. 10.3758/MC.37.4.477 19460954

[B33] Rudolph U. Schulz K. Tscharaktschiew N. (2013). Moral emotions: an analysis guided by Heider's naive action analysis. Int. J. Adv. Psychol. 2, 69–92. Available online at: https://bit.ly/3oHJAOI

[B34] Runco M. A. Jaeger G. J. (2012). The standard definition of creativity. Creat. Res. J. 24, 92–96. 10.1080/10400419.2012.650092

[B35] Stearns D. C. Parrott W. G. (2012). When feeling bad makes you look good: Guilt, shame, and person perception. Cogn. Emot. 26, 407–430. 10.1080/02699931.2012.675879 22471849

[B36] Storme M. Celik P. Myszkowski N. (2021). Creativity and unethicality: A systematic review and meta-analysis. Psychol. Aesthet. Creat. Arts 15, 664–672. 10.1037/aca0000332

[B37] Tang M. Li W. Q. Liu F. H. Yuan B. (2019). The association between guilt and prosocial behavior: a systematic review and meta-analysis. Adv. Psychol. Sci. 27, 773–788. 10.3724/SP.J.1042.2019.00773

[B38] Tangney J. P. Stuewig J. Mashek D. J. (2007). Moral emotions and moral behavior. Annu. Rev. Psychol. 58, 345–372. 10.1146/annurev.psych.56.091103.070145 16953797 PMC3083636

[B39] Xia X. T. Duan J. Y. Huang X. Y. (2021). FOMO's influence on prosocial behavior. Psychol. Dev. Educ. 37, 344–352. 10.16187/j.cnki.issn1001-4918.2021.03.05

[B40] Zhao J. Xu X. Pang W. (2022). When do creative people engage in malevolent behaviors? The moderating role of moral reasoning. Pers. Individ. Differ. 186, 111386. 10.1016/j.paid.2021.111386

[B41] Zhong C. B. Liljenquist K. (2006). Washing away your sins: threatened morality and physical cleansing. Science 313, 1451–1452. 10.1126/science.1130726 16960010

